# Supplementary material for: Developing a Mathematical Model of Intracellular Calcium Dynamics for Evaluating Combined Anticancer Effects of Afatinib and RP4010 in Esophageal Cancer
Source: Int J Mol Sci. 2022 Feb 3;23(3):1763. doi: 10.3390/ijms23031763 (PMC8836083; doi:10.3390/ijms23031763)
Supplement: Supplementary file 1 [file ijms-23-01763-s001.zip › ijms-1439589-supplementary.pdf]

Supplementary Materials

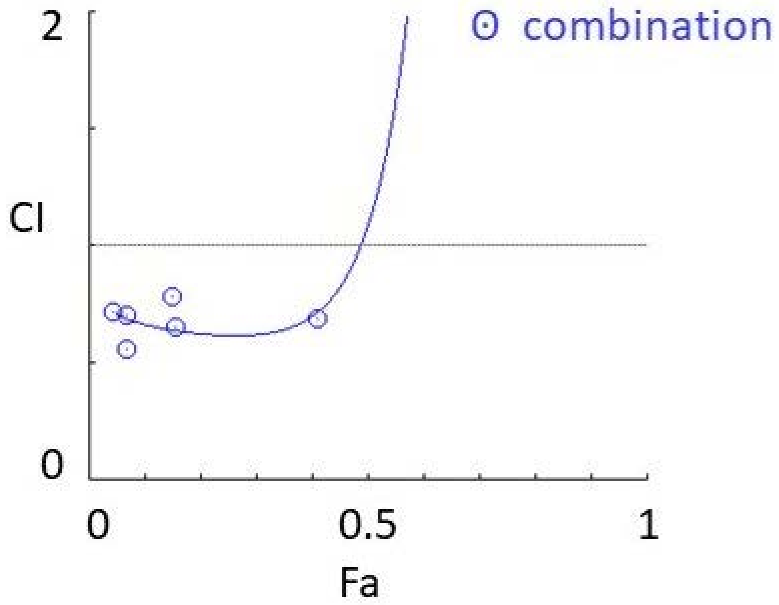

Figure S1. Combination Index (CI) Plot for combined effect of RP4010 and afatinib on the effect of Ca^{2+} oscillations in KYSE-150 cells. Each blue circle indicated a data point with combined RP4010 and afatinib. Simulation curve is showed in blue line. Average effect values (Fa) is showed in x-axis.

Table S1. Dose-dependent drug effects on intracellular Ca^{2+} oscillations in KYSE-70 cells (experimental data).

Drug concentration (μM)	Period (s) mean ± s.e.m	
	RP4010	Afatinib
5	68.7 ± 3.2	
2.5	58.0 ± 5.4	68 ± 5.3
1.25	49.9 ± 2.7	53.7 ± 1.5
0.625		51.9 ± 1.1
0.0	39.4 ± 1.5	

Table S2. Effects on intracellular Ca^{2+} oscillations by combined RP4010 and Afatinib in KYSE-70 cells (experimental data).

Concentration of Combined Drugs (μM)		Period (s)
RP4010	Afatinib	
5	2.5	75.5 ± 7.7
2.5	1.25	68.1 ± 4.5
1.25	0.625	59.2 ± 2.2
